# Sub-nanometer Resolution Imaging with Amplitude-modulation Atomic Force Microscopy in Liquid

**DOI:** 10.3791/54924

**Published:** 2016-12-20

**Authors:** Ethan J. Miller, William Trewby, Amir Farokh Payam, Luca Piantanida, Clodomiro Cafolla, Kislon Voïtchovsky

**Affiliations:** ^1^Physics Department, Durham University

**Keywords:** Engineering, Issue 118, atomic force microscopy, liquid, sub-nanometer resolution imaging, amplitude-modulation, lipid bilayer, biomembranes, crystals, cantilever, harmonics, eigenmode, solvation forces

## Abstract

Atomic force microscopy (AFM) has become a well-established technique for nanoscale imaging of samples in air and in liquid. Recent studies have shown that when operated in amplitude-modulation (tapping) mode, atomic or molecular-level resolution images can be achieved over a wide range of soft and hard samples in liquid. In these situations, small oscillation amplitudes (SAM-AFM) enhance the resolution by exploiting the solvated liquid at the surface of the sample. Although the technique has been successfully applied across fields as diverse as materials science, biology and biophysics and surface chemistry, obtaining high-resolution images in liquid can still remain challenging for novice users. This is partly due to the large number of variables to control and optimize such as the choice of cantilever, the sample preparation, and the correct manipulation of the imaging parameters. Here, we present a protocol for achieving high-resolution images of hard and soft samples in fluid using SAM-AFM on a commercial instrument. Our goal is to provide a step-by-step practical guide to achieving high-resolution images, including the cleaning and preparation of the apparatus and the sample, the choice of cantilever and optimization of the imaging parameters. For each step, we explain the scientific rationale behind our choices to facilitate the adaptation of the methodology to every user's specific system.

**Figure:**
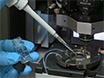


## Introduction

Since its invention, three decades ago, atomic force microscopy (AFM)^1^ has established itself as a technique of choice for investigating samples at the nanoscale, especially where averaging over macroscopic surface areas is not possible and local information is required. In a typical AFM measurement, the deflection of a flexible cantilever is used to quantify the interaction force between a small number of molecules and an ultrasharp tip mounted at the end of the cantilever. Depending on the type of interactions and the timescales considered, a wide range of information can be derived, including the viscoelastic properties of soft biological membranes^2,3^, the strength of a single chemical or molecular bond^4,5^, the atomistic details of a surface^6^^-^^8^, the magnetic^9^, capacitive^10^, conducting^11^, thermal^12,13^ and chemical^14^ properties of samples^15^. Part of the success of AFM is its ability to work on a wide range of materials^16^ and in multiple environments such as vacuum^17^, gas^11,18^ or liquid^19,20^, because it does not rely on a specific force between probe and sample.

In practice, however, operating the AFM in conditions other than ambient can be challenging and many published results are still obtained in air. An added difficulty comes from the fact that it is usually necessary to operate the AFM in dynamic mode (vibrating tip) in order to preserve both tip and sample by avoiding large friction forces. Although more challenging, dynamic operation can in principle provide more information about the sample analyzed, and with no loss of spatial resolution. Over the last decade, the field of dynamic AFM in liquid has seen important developments, from the advent of video-rate AFM^21^^-^^23^, to multifrequency measurements^24,25^ and sub-nanometer imaging of hydration structures at interfaces^26^^-^^31^. AFM operation while immersed in liquid is now routinely used in biology and biophysics^32^^-^^36^, polymer research^37^, electrochemistry^38^^-^^40^ and solid-liquid interfaces characterisation^41^^-^^44^. The presence of liquid around the vibrating cantilever considerably alters its dynamics^45^ as well as the interaction between the tip and the sample^29,42^. When used appropriately, the liquid can be exploited to enhance the imaging resolution^26,29^, with a typical improvement of almost an order of magnitude compared to the best resolution achieved in ambient conditions^46^.

In AFM, the highest spatial resolution achievable for a particular measurement depends on both the quality of the AFM itself and the nature of the interaction probed^20,47,48^. At the present time, most high-end, commercially available AFMs present noise levels that are close to that of thermal limit^12^ so the determining factor for resolution is usually the tip-sample interaction. It is effectively the spatial gradient of this interaction that determines the resolution: measurements based on short-range, rapidly decaying interaction produce higher resolution results than when longer-range interactions are at play. In liquid, solvation forces can improve imaging resolution because they tend to vanish over only a few molecular diameters of the liquid (typically < 1 nm) when moving away from the surface of the sample^49^. These forces originate from the interaction between the liquid molecules and the surface of the sample. A liquid with a strong affinity for the surface will tend to be more ordered and less mobile than bulk liquid at the interface with the sample^29,42,50^. As a result, it will take more energy for a vibrating AFM tip to displace interfacial liquid molecules than bulk liquid^42^, rendering the measurement highly sensitive to local variations in the interfacial liquid properties at the nanoscale -the solvation landscape.

In order to exploit solvation forces, several practical aspects need to be taken into consideration. First, the oscillation amplitude of the tip needs to be comparable to the range of the solvation forces, typically < 1 nm. Second, the liquid used must form a well-defined solvation landscape at the surface of the sample. Macroscopically, this is equivalent to requiring a 'wetting' liquid for the sample considered. For example, in water it is easier to achieve molecular-level resolution on hydrophilic mica than on hydrophobic graphite^42,51^. Finally, the spring constant of the cantilever supporting the tip must be selected appropriately^52,53^. When working in these conditions, the AFM does not only provide molecular-level images of the interface, but it also derives information about the local sample-liquid affinity which can be used to gain chemical information about the sample's surface^54^.

The most common dynamic modes of operation for AFM in liquid are amplitude-modulation (AM, also 'tapping mode') AFM and frequency-modulation (FM) AFM. In the first case^55^, the tip raster-scans the sample while its vibration amplitude is kept constant by a feedback loop that continuously re-adjusts the tip-sample distance. A topographic image of the sample is obtained from the correction applied by the feedback loop. In FM-AFM^28,41,56^, it is the oscillation frequency of the cantilever/tip that is kept constant while the tip scans the sample. Both techniques provide comparable topographic resolution in liquid^36,57^. Quantification of the tip-sample interaction tends to be more straightforward and accurate in FM-AFM, but AM-AFM is easier to implement, more robust, and allows working with softer cantilevers, something useful for studying easily deformable or delicate samples. Significantly, AM-AFM is more widespread among AFM users, partly for historical reasons but also due to the fact that it is technically easier to control.

Although the amplitude is kept constant by the feedback loop during AM-AFM imaging, the phase-lag between the tip oscillation and the driving oscillation is allowed to change freely. The phase-lag can provide useful information about the tip-sample interactions, being related to the energy dissipated during oscillation of the tip at the interface with the sample^58^. Hence phase-imaging can be acquired simultaneously to topographic imaging, and is often complementary in highlighting the heterogeneity of a sample surface. Phase imaging has been utilized for various mapping of interactions, including the direct mapping of adhesion energy^42^, viscoelastic properties^58^ and the hydration landscape of an interface^44^.

Practically, obtaining high-resolution images in liquid remains non-trivial due to the large number of parameters to control, and the absence of a simple, systematic protocol that works in every situation. Image quality typically depends on the cantilever geometry and elasticity, the tip chemistry, the oscillation amplitude, and the sample stiffness, among others^55^. AFM measurements are also, by definition, perturbative to the system. As a result, changing imaging variables and environmental conditions without proper considerations can lead to difficulties in reproducibility and/or misrepresentative observations and spurious results.

Here, we present our protocol for achieving high-resolution images of hard and soft samples in solution, using commercial instruments operated in amplitude-modulation. Our goal is to offer a practical procedure for optimizing the main parameters likely to influence the resolution over different samples, explaining in each case the rationale for our choices from the physical principles underlying the imaging process. We detail a step-by-step approach, from substrate cleaning and preparation, to choice of cantilever, adjustment of the imaging parameters and troubleshooting common problems. Explaining the scientific rationale behind our choices and procedures for high-resolution should help making rational choices when adapting the methodology, and serve as a starting point for imaging novel systems.

Throughout this text we use AM to refer to the amplitude-modulation operation mode of an AFM. We refer to the feedback parameter kept constant during either the cantilever deflection (contact mode) or oscillation amplitude (AM mode) as the *setpoint* value. In AM mode, the cantilever is externally driven either by an acoustic oscillation or by a pulsed laser focused at the base of the cantilever. The *drive amplitude* is the intensity of the external oscillatory signal. The absolute value of the drive amplitude required to achieve a given oscillation amplitude of the cantilever depends on many parameters such as the method of driving (acoustic, photothermal or magnetically), cantilever fixation and parameters (stiffness, geometry) and laser alignment. The exact value of the drive amplitude is therefore not relevant but it is adjusted in each experiment so as to provide an appropriate (and quantifiable) oscillation amplitude of the cantilever. When the driven cantilever is far away from the sample and no damping of its vibration occurs through tip-sample interactions, its oscillation amplitude is called the *free oscillation amplitude*. As the vibrating tip nears the surface of the sample, its amplitude starts to decrease. If the feedback is enabled, the z-piezo will constantly re-adjust its extension so that to keel the selected setpoint amplitude, constant. The setpoint value is normally always smaller than the free amplitude. It is common to refer to the *setpoint ratio*, the ratio of the setpoint amplitude (imaging amplitude) over the free amplitude. The smaller the setpoint ratio, the harsher the imaging conditions are.

## Protocol

### 1. Cleaning of Tools and Other Surfaces

NOTE: When aiming for high resolution, any form of contamination can have detrimental consequences. It is therefore necessary to ensure all the tools used to manipulate the sample, substrate or AFM tips are thoroughly cleaned. The following applies to any surface or instrument (*e.g.,* tweezers) that may come into contact with the sample, cantilever, or AFM cell, including the sample stage itself.

1. Bath-sonicate the instruments in ultrapure water (18.2 MΩ, <5 ppm organics), followed by isopropanol (99.7% purity), followed by again ultrapure water, each for 10 min. When possible, use isopropanol under a fume hood to reduce inhalation of fumes.
2. Dry under a nitrogen flow.
3. If full immersion is not possible (*e.g.,* for cantilever holder/electronics), physically clean the surface by wiping with single-ply, low-lint tissues (light-duty tissues wipers) soaked in ultrapure water, isopropanol and ultrapure water, sequentially. Allow the surface to dry in air (usually within 15 to 30 min).

### 2. Substrate Preparation

NOTE: The substrate designates the solid surface directly supporting the samples, typically in physical contact with both the AFM scanner and the sample. Most AFMs have a magnetic mount and steel disks can be used, but the same protocol is also suitable for substrates such as glass slides. Here we assume a steel disk on which a mica disk is affixed. The goal of this procedure is to limit as much as possible external sources of contamination that can affect the imaging. Gloves must be worn at all times.

1. Bath-sonicate the steel sample disc in ultrapure water (18.2 MΩ), followed by isopropanol, and finally by ultrapure water again, each for 10 min.
2. Dry the discs under a nitrogen flow.
3. Prepare a small amount of epoxy glue with reagents mixed thoroughly and place ~10 µl on the steel disc.
4. Affix the substrate (muscovite mica, SiO_2_ crystal, glass, *etc.*) to the steel disc by applying pressure on the substrate. Allow the epoxy to cure for several hours at an elevated temperature (see manufacturer's specifications).
5. Ensure that no epoxy is directly exposed to the air, around the edges of the substrate. This will happen if excessive amount of epoxy is used and may become a source of contamination.
  1. For a mica substrate, firmly press ~2.5 cm wide adhesive tape onto the substrate, so that the entire face is covered, and smoothly peel it off. Use mildly adhesive tape, and peel the mica by pulling parallel to the surface. The removed material is visible on the tape. Repeat this process 2-3 times, until the mica is mirror-smooth to the eye.
  2. For glass/SiO_2_, if further chemical surface modification is required, repeat the bath-sonication routine of steps 2.1-2.2. Alternatively, use UV exposure unit (18 W UV-C germicidal lamps) for 30-60 min, depending on power) to pyrolyze any organics that may be left on the surface. This procedure also renders the surface more hydrophilic without significantly increasing roughness.

### 3. Preparation of Cantilever and Tip

1. Immerse the cantilever chip in a bath of isopropanol, followed by ultrapure water, each for 60 min.
2. If the cantilever/tip requires extensive cleaning (*e.g.*, after prolonged storage in gel box), add a 30 min soaking in acetone (> 99.5% purity) before step 1 (see also the section addressing contamination). The storage of tips in gel boxes is, in our experience, one of the primary source of contamination that can occur very rapidly^59^.
3. Expose the tip to UV light briefly (<5 min) in order to favor the formation of stable hydration sites^60^. Avoid longer overexposure times as it can damage the tip or increase its radius of curvature.
4. Insert the cantilever into the AFM's cantilever holder and pipette ~50 µl of imaging solution (the nature of the solution will depend on the sample being investigated, but in this case use a 10 mM solution of rubidium chloride in ultrapure water) onto the cantilever and tip to pre-wet it; this will limit the appearance of air bubbles when approaching the sample.

### 4. Set-up of AFM Cell

1. Mount the sample disc and substrate onto the sample stage and add a droplet of imaging liquid (liquid typically 2-3 mm thick at its highest point).
2. Connect the cantilever holder to the AFM.
3. Bring the cantilever and sample into close proximity so as to form a capillary bridge between the fluids on the cantilever/tip and the sample.

### 5. Initializing Measurement and Calibration of Cantilever

1. Align the measuring laser (usually red) close to the tip-end of the cantilever. Depending on the AFM model, do so either via software controls or by manual adjustment of the laser position. Acquire the cantilever's thermal spectrum in liquid (see **Figure 1A**). The process records the thermal fluctuations of the cantilever using the laser, in order to find the frequency of the cantilever's main resonance (fundamental eigenmode). In most modern AFM, this is done through automated procedures in the software controls, but details may vary from AFM to AFM.

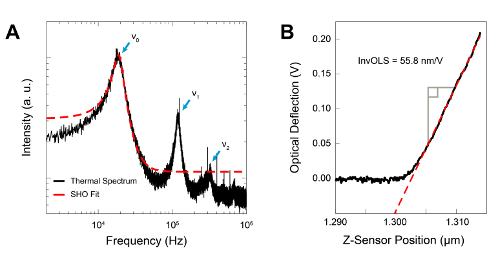
**Figure 1: Tuning and calibrating the cantilever. A**: Thermal noise spectrum of the cantilever's vertical deflection (black) with a simple harmonic oscillator (SHO) fit of the fundamental eigenmode (red). Here the resonance is 18.7 kHz in water. The first three resonance frequencies which correspond to the first three flexural eigenmodes are highlighted with blue arrows. **B**: Calibration of the cantilever's Inverse Optical Lever Sensitivity. The linear deflection of the cantilever pressed against a rigid (non-deformable) surface is used to measure the conversion factor (InvOLS) between the deflection measured in volts and the corresponding value in nanometers. Please click here to view a larger version of this figure.

1. Record a deflection vs. distance curve with the cantilever on a stiff substrate (*e.g.*, mica or glass) and calibrate the deflection by imposing that the slope of the curve in the linear deflection region (as in **Figure 1B**) is unity^61^^-^^63^. NOTE: This process finds the conversion factor between the measured deflection on the photodetector (in volts) and the real deflection of the cantilever (in nanometers) — known as the Inverse Optical Lever Sensitivity (InvOLS). The calibration may damage the tip, so it is better to conduct it after all measurements are completed. Use the InvOLS value derived for another cantilever of the same type during a previous experiment to get an estimate (typically >90% accurate) of the true amplitude before calibration.
2. Fit the resonance peak in the thermal spectrum with a simple harmonic oscillator model^64^^-^^66^ to yield the spring constant of the cantilever. This procedure is automated in most AFM software and does not usually require specialist knowledge of the model in question.
3. Tune the cantilever. Find the amplitude response of the cantilever when externally driven (*e.g.,* acoustically or by photothermal excitation) over a range of frequencies close to its nominal resonance frequency. Set the driving frequency to the near the maximum in this spectrum, slightly to the left. If the cantilever is acoustically driven, many spurious maxima may appear when tuning.
  1. Select a resonance peak as close as possible to (and within the envelope of) the resonance identified in the thermal spectrum by using the control software of the AFM but specifics may depend on the software type and version that controls the AFM.

### 6. Approach and Initial Check of Sample

1. Set the driving amplitude so that the free oscillation amplitude is approximately 5 nm. This typically corresponds to 0.2-0.8 V on most AFMs (in case the InvOLS is not calibrated). Adjust the amplitude setpoint to ~80% of the free amplitude.
2. Set the feedback gains relatively high (the absolute value depends on the AFM) but ensure that no instability or ringing occurs.
3. Set the initial scan rate and scan size to small values (*e.g.*, to ~1 Hz and 10 nm respectively). This helps preserve the tip in case some feedback parameters are poorly adjusted by avoiding scanning over large distances. The scan size can be subsequently increased to a larger value (*e.g.,* 100 nm) if the scanning conditions appear suitable.
4. Determine the approximate height of the surface (in some cases this must be done optically) before the approach.
5. Initiate the tip's approach to the surface using the AFM control software. The fine details of this process will depend on the model of AFM and software used.
  1. If there are problems with the approach when using a soft cantilever, conduct the approach in contact mode. In this case, ensure that the gains are lower than in AM mode, and set the setpoint to a relatively low value (0.1-0.2 V after centering the laser on the photodetector) to preserve the tip.
  2. Assess whether the tip has reached the surface without starting to image by slightly changing the setpoint value (increase in contact mode or decrease in AM mode). If the tip is at the surface, the effect on the extension of the Z-piezo should be negligible. The live motions of the Z-piezo are usually displayed graphically in the control software of most AFM. If the setpoint change triggers a visible motion of the piezo, this indicates a false engage. In the latter case, re-start the approach from the current tip position, using a slightly higher (contact) or lower (AM) setpoint.
6. Once the tip has reached the surface, retract the Z-piezo (usually by simply pressing 'stop') and retune the cantilever (repeat step 5.4.); the resonant frequency will likely have shifted to a lower value due to tip-sample interactions.
7. Change the set point to ~80% of the newly tuned free amplitude (at this tip-sample distance).
8. Engage the cantilever and conduct a 10×10 nm^2^ scan of the surface in AM mode to verify that the imaging parameters are suitable.
  1. Check that the trace (scanning left to right) and retrace (scanning right to left) profiles superimpose. If not further reduce the setpoint and try increasing the gain.
  2. Lower the gains if the image becomes noisy.
9. Repeat the operation with a large — 1×1 µm^2^ to 5×5 µm^2^ — scan of the sample provided this is possible. On soft or biological samples, this might result a contamination of the tip.

### 7. High-resolution Imaging

1. Reduce the scan size to a value suitable for visualizing the features (*e.g.*, 100×100 nm^2^ for protein crystals or 20×20 nm^2^ for mica or calcite).
2. Reduce the drive amplitude of the cantilever enough for the feedback loop to automatically retract the Z-piezo and hence the tip from the surface. While the cantilever is away from the surface, adjust the drive amplitude so that the cantilever amplitude is ~1-2 nm (peak-to-peak).
3. Using the AFM control software, progressively reduce the setpoint a few tens of mV at a time until the Z-piezo extends again towards the surface and the original image is recovered. Keep the setpoint amplitude between 75% and 95% of the new free amplitude.
4. Readjust the gains using the AFM control software; higher gains can be used at lower amplitudes without introducing significant noise.
  1. Repeat the steps 7.2-7.4 so as to determine the best combination of free amplitude, setpoint and gain for high-resolution. The optimum conditions depend on the sample (solvation landscape and wetting properties of the liquid) but also the cantilever (spring constant, stiffness).
  2. Explore different combinations of amplitudes to optimize imaging conditions. It may be necessary to increase again the free amplitude^42^. In such a case, adjust first the setpoint to a higher value and then increase the drive (*i.e.*, reverse procedure than used to decrease the amplitude).
  3. Keep the setpoint amplitude in the range of 0.5 nm - 1.5 nm (peak to peak) with setpoint ratios kept above 0.7 (typically 0.75-0.95). For solvophilic interfaces, use cantilevers with a spring constant of 0.5 - 2 N/m. This is sufficient for the tip to remove most of the interfacial liquid without hitting the surface. A rule of thumb is proposed in eq. 4 of reference^29^.

## Representative Results

The protocol described in the previous section has been successfully applied with several commercial AFMs to achieve molecular- or atomic-level images. All images were obtained with working amplitudes between 0.5 nm and 1.5 nm adjusted individually following the procedure steps 7.1-7.4. Results could be obtained over a broad range of soft (**Figure 2**) and stiff (**Figure 3**) samples. In each case, the features of interest are highlighted. One of the great advantages of the technique is that small oscillation amplitudes and high set-points minimize the force exerted by the tip on the sample, allowing fragile self-assemblies of lipids (**Figure 2A**), proteins (**Figure 2B **and** D**), and amphiphilic molecules (**Figure 2C**) to be imaged without damage in solution. Harder crystalline materials such as minerals (**Figures 3A, B, D**) and single metal ions adsorbed on a surface (**Figure 3C**) can be imaged using the approach because in every case, it is the interfacial liquid that is effectively imaged with the protocol described. The solvation forces are comparable on the samples shown **Figures 2 **and** 3**: all samples are hydrophilic (more generally, "solvophilic" in the case of **Figures 2C, 3B, 3D**) with respect to the imaging solution. Consistently, cantilevers with comparable stiffness (0.2 - 0.8 N/m) were used in all cases. Both the sample and the tip should be solvophilic to ensure that liquid molecules form a well-defined solvation structure that can be imaged. This is not always a sufficient condition, but in most cases and for relatively small liquid molecules, the liquid re-structures itself in a way that mimics the sample symmetry. The main driver of the high-resolution is the local variation in the affinity of the adsorbed solvent molecules for the surfaces (Ångström-scale, in the case of **Figures 2A, 3A, 3C**). The technique is therefore best suited to materials where the solvent structure varies to a great extent across the surface.


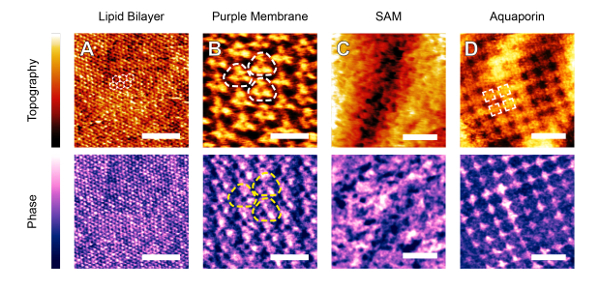
**Figure 2: Soft interfaces imaged by AM-AFM in aqueous solutions. A**: Dipalmitoyl-phosphatidylcholine (DPPC) lipid bilayer in gel phase imaged in in 150 mM KCl. The hexagonally-packed lipid headgroups are distinguishable in both topography and phase, along with local contrast indicating site-specific variations in hydration. **B**: Purple membranes of *Halobacterium salinarum* imaged in 150 mM KCl, 10 mM Tris, pH 7.4. Several bacteriorhodopsin protein trimers are highlighted. The phase exhibits a contrast different from topography due to local hydration sites on the proteins. Individual protein trimers can be made out (dashed lines). **C**: Amphiphilic dye (Z907) molecules adsorbed at the surface of a TiO_2_ nanoparticle in a dye-sensitized solar cell. The image was acquired in ethyl-isopropyl sulfone. The sponge-like appearance is created by the adsorbed dye molecules. **D**: Aquaporin crystal in native bovine lens membranes imaged in the same buffer as B. An aquaporin tetramer is highlighted. Sub-structure corresponding to inter-helical loops are visible in topography while phase shows a strikingly different contrast due to the unusual water behavior near the protein. The images are adapted from refs^36^ (B), ref^38^ (C) and ref^67^ (D). The scale bar is 5 nm (A), 10 nm (B), 3 nm in (C), and 15 nm (D) The color scale indicates respectively a height and phase variation of 200 pm and 15° (A), 600 pm and 4° (B), 2.5 nm and 2.5° (C), and 1.6 nm and 9.5° (D). Please click here to view a larger version of this figure.


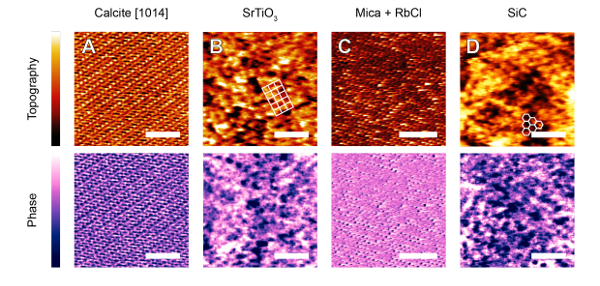
**Figure 3: Representative AM-AFM images of hard samples in fluid solutions. A**: Calcite crystal [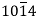] surface imaged in an equilibrated ultrapure water solution. **B**: Strontium titanate imaged obtained in dimethyl sulfoxide (DMSO). High-resolution was not possible in water. **C**: Muscovite mica imaged in 3 mM RbCl — single adsorbed Rb^+^ ions are visible on the mica's lattice sites in both phase and height scans. **D**: Silicon carbide in imaged in DMSO. The expected crystallographic arrangements are shown in images B and D. The images are adapted from ref ^68^ (A), ref ^42^ (B and D), and ref ^44^ (C). The scale bar is 3 nm (A, B, D) and 5 nm (C). The color scale indicates respectively a height and phase variation of 250 pm and 14° (A), 600 pm and 5.5° (B), 800 pm and 15° (C), and 500 pm and 3.5° (D). Please click here to view a larger version of this figure.

## Discussion

Assuming that the imaging liquid and the cantilever stiffness have been selected appropriately, the most critical steps for achieving successful high-resolution are the adjustment of the imaging amplitude, and the overall cleanliness of the system investigated.

Amplitudes comparable to the thickness of the interfacial liquid region (typically less than 2 nm) probes mainly variation in the properties of the interfacial solvent^42^. If the oscillation amplitude is too large, the vibrating tip will traverse long-range, non-linear force fields^52^ that preclude the stability of cantilever motion, and inevitably hit the sample regardless of the imaging conditions^29^, resulting in deterioration of the resolution. Aside from the loss in resolution, higher harmonics start to appear in the tip motion and the system becomes more complicated to model^55^. Alternatively, if the imaging amplitude is too small only part of the interface is probed (typically specific layers of the interfacial liquid) and stable imaging can only be achieved with stiff cantilevers (>10 N/m in water^53^) for a satisfactory signal-to-noise ratio, with the risk of damaging soft samples over large height variations. The need for stiff cantilevers is to overcome the thermal noise that can become more significant that the signal measured When working with small amplitudes, the long-range interactions between the tip and the sample are still present, but are largely constant and do not affect the high-resolution contrast in the images obtained.

Cleanliness of the imaging environment is of paramount importance when it comes to high-resolution AFM. Undesired compounds in the system can interfere with both the imaging and the force spectroscopy. There are two main categories of contaminations that tend to affect the experiments: (i) contaminants directly visible when imaging (**Figure 4B, 4C**) and (ii) general unexplained lack of high-resolution. Case (i) tends to occur only in highly idealized systems such as at the water-mica interface where adsorbed molecular aggregates that interfere with the tip-sample interactions are clearly contrasted against the atomically flat mica surface (**Figure 4A**). Before changing the tip and the sample, it is worth acquiring spectroscopic curves with a large deflection, effectively pressing hard the tip against the sample repeatedly. This would normally damage a new tip, but can occasionally clean a dirty tip or induce stable hydration sites suitable for imaging. This tip will, however, inevitably be blunted and hence be only suitable for flat sample even if the imaging improves. In case of suspected contamination over stiff samples, it may be worth trying to image with the second eigenmode of the cantilever before attempting the somewhat destructive procedure described above. This simply requires switching the driving frequency to the second eigenmode and readjusting the amplitude/setpoint (see the troubleshooting discussion below). The effective stiffness of the cantilever increases considerably when operated at the second eigenmode and any weakly adsorbed contaminant may be pushed away by the tip while imaging. This strategy does not replace the need for a clean sample and tip, but offers some further avenues to acquire satisfactory images when a tip/sample is clearly not ideal.


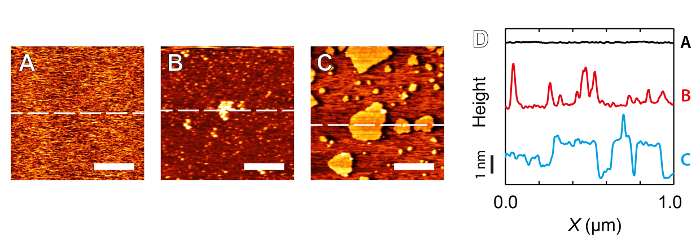
**Figure 4: Examples of contamination observed when imaging muscovite mica that inhibit high-resolution imaging. A**: Mica imaged in 5 mM RbCl — no contaminant particles are visible. **B**: Contamination taking the form of aggregates of the order of tens of nm across while imaging in nominally ultrapure water. **C**: Self-assembled structures formed by contaminant particles presumably amphiphilic in nature. Imaging was again conducted in nominally ultrapure water. **D**: Vertically offset sections corresponding to the dashed lines in A, B and C illustrating the deviation from mica's atomically flat surface. Scale bars in A, B and C correspond to 300 nm. Please click here to view a larger version of this figure.

Case (ii) is more common and characterized mainly by the frustrating fact that sub-nanometer features simply cannot be resolved, regardless of the imaging conditions. The signature of this type of situation is usually visible in force spectroscopy measurement which tend to show some inconsistencies. These may include poorly reproducible curves and amplitude vs distance curves that significantly deviate from a typical sigmoidal shape^42^. If contaminants, ionic or otherwise, are dispersed homogeneously throughout the fluid, they may not show up in topographic imaging but could disrupt the hydration structure of the sample^69^, which is crucial for maintaining a regular tip-sample interaction^29^ and obtaining high resolution^70^. There may also be direct effects of the contaminants on the sample, especially in soft, biological experiments. For example, it is well known that the presence of alcohols (from the cleaning procedure) can fluidify gel-phase lipid bilayers^71^^-^^73^, rendering sub-nanometer level resolution impossible. If high-resolution is not possible, care should be first taken in the cleaning process, focusing especially on any equipment that comes into contact with the imaging solution. Even ostensibly stable compounds such as the epoxy resin may solvate in the fluid to some extent if not fully cured.

High-resolution imaging with AM-AFM is demanding, requires patience and often several trials before reaching the best possible imaging conditions. Small experimental issues can easily become important enough to prevent high-resolution and troubleshooting skills are essential. Hereafter we list some of the most common problems we encountered with our proposed solution.

### Cantilever tuning

Most commercial AFMs use acoustic excitation to drive the cantilever. In such case, tuning the cantilever, as described in step 5.4, near its resonant frequency often provides sufficient performance for operation in air. In liquid environments, the liquid tends to induce some coupling between different mechanical parts of the AFM such as cantilever chip and the holder. This can affect the apparent resonance of the cantilever, often illustrated by a cantilever frequency spectrum that exhibits many sharp peaks and valleys commonly described as a "forest of peaks". As a result, it is often difficult to find the correct drive frequency. These peaks also exist in gas environments, but due to the high value of quality factor of cantilever, the amplitude at resonances is considerably larger^74,75^. In liquid selecting the appropriate peak to drive the cantilever may be not easy and can require trial and error. In practice, the frequency peak with steepest variation in amplitude in the "forest of peaks" around the resonance frequency is usually the best bet despite being not necessarily exactly on resonance and often provides a driving frequency adequate to obtain high-resolution imaging.

### Image distortion

Imaging drift is often an issue when seeking high-resolution and makes the images look distorted (typically stretched). Its origin is generally thermal, either because the scanner/AFM has not reached its equilibrium operating temperature, or because part of the sample liquid is evaporating rapidly (*e.g.*, imaging in alcohols). In all cases, the drift becomes negligible at thermal equilibrium. It is therefore useful to fix the temperature of the sample if possible. Otherwise, it is worth leaving the AFM to scan a blank sample (large size scan at slow scan rate) for several hours before conducting the experiment. If evaporation is not an issue, this procedure is best done after step 6 of the procedure, taking care to first withdraw the tip a short distance (*e.g.*, 20 µm) from the surface. Occasionally, the drift will remain even after extensive thermalization. This usually indicates that the cantilever or its chip is partly dragging the sample while imaging, something that can happen on soft cohesive samples such as thin films or if the tip/cantilever/chip is not suitably placed. On chips that host more than one cantilever/tip, it is often helpful to break the cantilever that are not in use rather than let them drag over the surface.

### Ionic strength

Since the imaging is dominated by the interfacial liquid, it is sometimes helpful to add some salt for high-resolution imaging of the charged surface in water. The role of the salt is twofold. Firstly, it modifies the hydration landscape of the surface imaged upon adsorption, which often enhances the contrast. Secondly, it helps screen strong electrostatic interactions between the tip and sample (*e.g.,* on mica). Generally, larger ions such potassium, rubidium and cesium allow better images due to their specific hydration properties^76^, and the fact that they often adsorb mainly in a unique hydration state^77^.

### Bad cantilever/tip

If it is suspected that the cantilever is a source of contamination (see symptoms described above), it should be first inspected under an optical microscope. If stored in a gel box, the cantilever may pick up traces of gel polymers or silicon oil^59^ that can appear, in extreme cases, as darker spots, on the back of the cantilever (as in **Figure 5A**). Photothermal oscillation of the cantilever can induce similar spots, but they are due to degradation/overheating of the cantilever coating by the driving laser. Contamination tends to appears randomly on the cantilever. A longer (12 hr) cleaning with isopropanol and, then, with ultrapure water can remove any undesired particles from the cantilever.


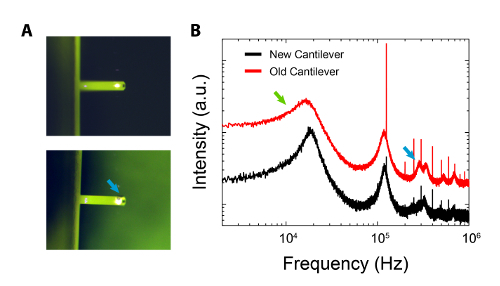
**Figure 5: Comparison between a new cantilever and an identical one that has been used extensively on hard surfaces and left in a gel box for an extended period. A**: Top; optical image of brand-new cantilever that has been cleaned (see procedure). Bottom; optical image demonstrating the appearance of visible contamination (blue arrow) from gel box. **B**: Comparison of cantilevers' respective thermal spectra. Broadening of the old cantilever's first resonance peak is clear (green arrow) and some higher-order modes are enhanced (blue arrow). Spectra have been vertically offset and presented on a log-log scale for clarity. Please click here to view a larger version of this figure.

If the required sub-nanometer resolution is not achieved, despite acceptable images at lower resolution, it is possible that the AFM tip has become chemically modified during its storage environment. This can be treated by exposure of the cantilever chip to an ultraviolet oxidizer for 120 sec, which aids the creation of hydrophilic surface groups on the tip^60^. Care should be taken however, as the exact time necessary may vary depending on tip geometry and UV power, and over-exposure may result in blunting of the tip and reduced resolution.

### Thermal noise

High-resolution imaging requires great sensitivity to variations in force and distances (typically sub-pN forces and sub-Ångström distances^78^). For softer cantilevers, the thermo-mechanical motion of the cantilever due to its intrinsic Brownian motion (thermal vibration) can be a problem. In first approximation, with a cantilever of stiffness *k*, it is not possible to measure features smaller than 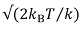, the amplitude of the thermal noise, where *k_B_* is Boltzmann constant and *T* is the temperature. Practically, using cantilever with higher resonance frequencies spreads the noise over a larger frequency range, and reduces the overall noise level in the measurement bandwidth^79^.

### Higher eigenmode imaging

It can sometimes be useful to operate the cantilever at its second eigenmode due to the increase effective stiffness (see discussion of contamination). Practically, this is done simply by driving the cantilever at its second eigenmode (the second resonance peak at higher frequency, see **Figure 1A**). When tuning the cantilever, simply select the second eigenmode instead of the main resonance and proceed to step 5.4. Note that the InvOLS will be different when the cantilever is driven at the second eigenmode; typically ~1/3 of the InvOLS measured in step 5.2 for a rectangular cantilever.

The main limitation of the technique is that it requires a stable solvation landscape at the surface of the sample. The sample should be robust enough to allow perturbing the interfacial liquid without inducing significant deformation of the sample itself. This can be challenging on very soft and unstable samples such large biomolecules. Additionally, small-amplitude AFM as described here cannot obtain mechanical information about the properties of a sample, as the cantilever tip spends the majority of its time in the interfacial fluid. For this, it may be beneficial to use other approaches such as Quantitative Nanomechanical Mapping^80^ or make use of higher harmonics of cantilever motion. Higher harmonicas are generally enhanced when imaging in fluid (with low quality-factors)^29,81^^-^^83^ and can provide simultaneously topography and stiffness of samples^25,81^^-^^84^ but they are generally detrimental to high resolution. Other limitations inherent to all scanning probe microscopy techniques are still valid here, in particular the fact that the results inevitably contain information about the measuring tip. The use of small amplitudes is also not ideal for samples with large height variations; the feedback loop will inevitably react more slowly when height variations are larger than the imaging amplitude, hence risking sample and tip damage. The use of softer cantilever mitigates this problem to a certain extent.

The main advantage of the method presented here is the fact that it provides the highest image resolution possible with AFM in liquid but can be implemented on any commercial AFM, provided that the noise levels of the machine are low enough. Comparable resolution on commercial instruments is usually achieved in contact mode, or occasionally in FM-AFM with stiff cantilevers. Working in AM-mode and with relatively soft cantilevers allows for a broader choice of samples, and is easier to implement than FM-AFM on most systems. The approach relies on exploiting the solvation forces existing at the interface between any solid and liquid to enhance resolution and gain local chemical information. It can in principle be used in ambient conditions, relying only on the water layers (typically several nanometers thick) building up on most surfaces due to the air's humidity. The principles underlying the high-resolution strategy remain unchanged but most of the tip is in air, with only a capillary bridge between the tip apex and the sample^85^. High-resolution has been demonstrated on stiff samples in these conditions^86,87^. The imaging conditions are however different than those of immersed liquid due to a higher Q-factor of the cantilever's oscillation. Practically, we found it difficult achieve stable operation over soft or irregular samples, presumably due to temporal changes of the capillary bridge and increased Q-factors for a given cantilever stiffness.

The protocol described herein offers a methodology for achieving molecular-level resolution images of samples in liquid with most modern commercial AM-AFMs. We provide the scientific rationale behind our choice of imaging parameters and emphasize the role of solvation forces. We also discuss common problems and in particular contamination. The specific tip-sample interactions can vary dramatically depending on the content of the imaging solution, the cantilever geometry and material, and the sample chemistry. A practical understanding of the nature of the dominant forces present during scanning is therefore essential to adapt this protocol to new systems and ensure reliable results. When optimized, the experimental approach is powerful to gain *in-situ* local molecular level insights of samples in solution.

## Disclosures

The authors have nothing to disclose.
